# Quick-Release Antifouling Hydrogels for Solar-Driven Water Purification

**DOI:** 10.1021/acscentsci.2c01245

**Published:** 2023-02-08

**Authors:** Xiaohui Xu, Néhémie Guillomaitre, Kofi S. S. Christie, R. Ko̅nane Bay, Navid Bizmark, Sujit S. Datta, Zhiyong Jason Ren, Rodney D. Priestley

**Affiliations:** ^†^Department of Chemical and Biological Engineering, ^‡^Department of Mechanical and Aerospace Engineering, ^§^Princeton Materials Institute, ^∥^Department of Civil and Environmental Engineering, and ^⊥^Andlinger Center for Energy and the Environment, Princeton University, Princeton, New Jersey 08540, United States

## Abstract

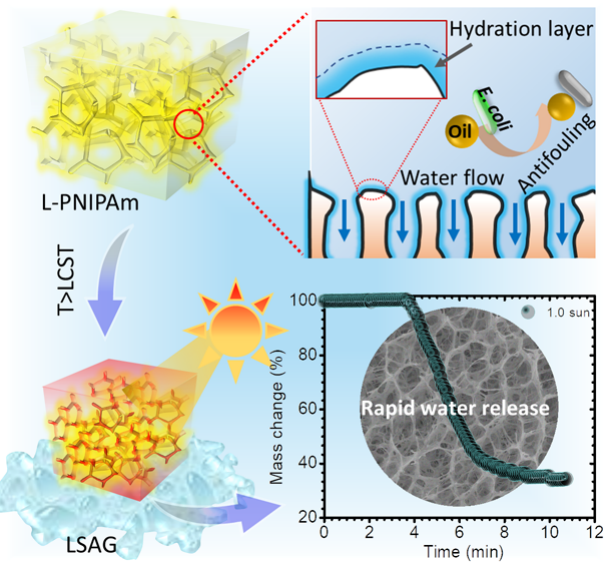

Hydrogels are promising soft materials for energy and environmental applications, including sustainable and off-grid water purification and harvesting. A current impediment to technology translation is the low water production rate well below daily human demand. To overcome this challenge, we designed a rapid-response, antifouling, loofah-inspired solar absorber gel (LSAG) capable of producing potable water from various contaminated sources at a rate of ∼26 kg m^–2^ h^–1^, which is sufficient to meet daily water demand. The LSAG—produced at room temperature via aqueous processing using an ethylene glycol (EG)–water mixture—uniquely integrates the attributes of poly(*N*-isopropylacrylamide) (PNIPAm), polydopamine (PDA), and poly(sulfobetaine methacrylate) (PSBMA) to enable off-grid water purification with enhanced photothermal response and the capacity to prevent oil fouling and biofouling. The use of the EG–water mixture was critical to forming the loofah-like structure with enhanced water transport. Remarkably, under sunlight irradiations of 1 and 0.5 sun, the LSAG required only 10 and 20 min to release ∼70% of its stored liquid water, respectively. Equally important, we demonstrate the ability of LSAG to purify water from various harmful sources, including those containing small molecules, oils, metals, and microplastics.

## Abstract 2

An open-cell gel-based membrane prepared using the mixed-solvency effect with photothermal and antifouling capabilities enables the rapid purification of water from contaminated sources.

## Introduction

Providing access to safe water is a pressing global challenge due to the expansion of industrialization, growth of the worldwide population, and contamination of freshwater resources.^1^ According to the United Nations, in the last century, global water demand grew more than twice that of the population growth rate.^2^ The Environmental Protection Agency (EPA) has identified over 70000 water bodies in the United States alone that are impaired by pollution.^3^ Currently, ∼4.5 billion people live near impaired water sources, and ∼52% of the world’s population will live in a water-stressed region by 2050.^4^ The health issues associated with consuming contaminated water are well-known: waterborne disease outbreaks that lead to gastrointestinal illness, reproductive complications, and neurological disorders, among others. More than 1.5 million people die each year from diarrhea caused by the intake of unsafe drinking water.^5^ In addition, overcoming the current COVID-19 pandemic and preventing future ones require access to clean water for sanitation purposes. Therefore, developing advanced water purification technologies that provide access to safe and clean water to more of the global population, especially those in under-resourced environments, remains an enduring challenge.

Promising approaches to alleviating the water scarcity problem require ease of manufacturing and operation that are advantageous for technology implementation. Also, energy-saving technologies are desired to achieve a balance between energy consumption and water production.^6^ To this end, the solar-driven water evaporation strategy is desirable for sustainable freshwater production because of the abundance of solar energy.^7−10^ New evaporation-based technologies seek to overcome the intensive solar energy requirement that results in a relatively low water evaporation rate under natural sunlight.^11−14^ These approaches include heat localization at the air–water interface to reduce heat loss and new materials for broad solar absorption. Yet, the need to overcome the heat of vaporization limits the production rate.

One approach to reducing the energy needed for sustainable water production is to use thermoresponsive hydrogels, specifically poly(*N*-isopropylacrylamide) (PNIPAm), which exhibits an accessible lower critical solution temperature (LCST).^15−24^ Near the LCST at ∼33 °C, PNIPAm-based hydrogels can absorb and release liquid water via hydrophilic/hydrophobic switching. The low LCST for water release—a temperature readily achievable using natural sunlight as the heating source—distinguishes PNIPAm from other materials requiring high energy consumption.^25−27^ PNIPAm-based technologies have shown promise in wastewater purification,^28−31^ desalination,^32−34^ and moisture harvesting.^35−38^ Nevertheless, conventional PNIPAm (C-PNIPAm), characterized by a closed-pore structure, suffers from a slow response rate above the LCST due to the formation of a dense skin layer, which acts as a barrier that entraps absorbed water and reduces the water release rate.^39^ Thus, current solar-driven hydrogel-based water purification systems can produce only a few gallons of water per day, well below the recommended use of ∼15–40 gallons per person.^40^ Furthermore, fouling at the surface caused by oil, biologics, and other pollutants during operation is still a significant obstacle for long-term water purification from polluted water resources for this technology.^41,42^ Therefore, overcoming these barriers by developing rapid-response and antifouling hydrogels is critical for establishing thermoresponsive hydrogel systems as a future commercial technology to improve clean water access.

**Figure 1:**
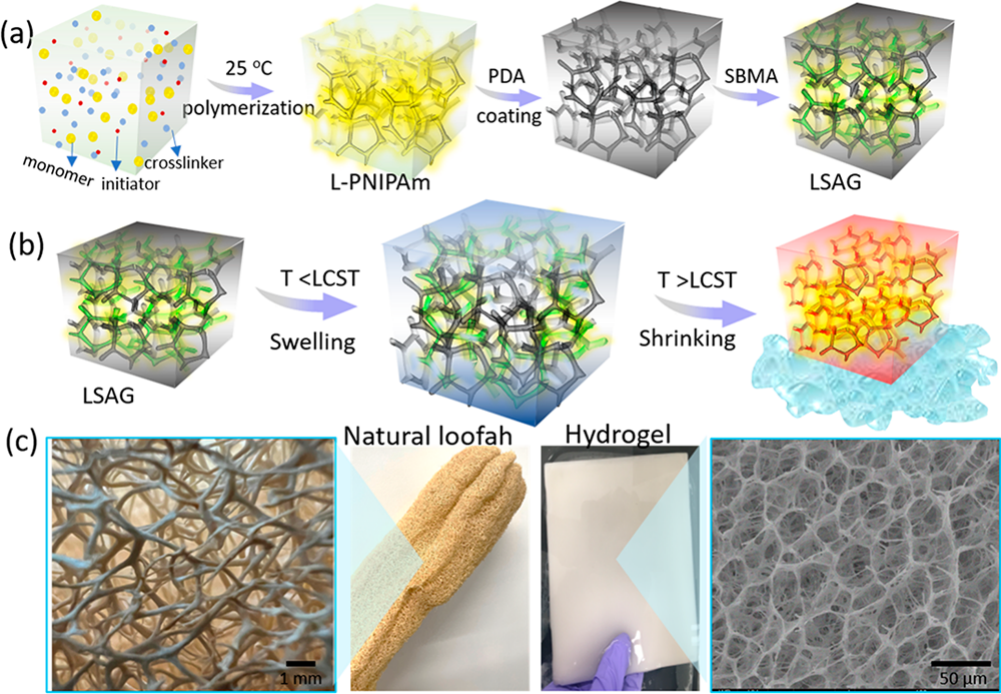
Fabrication and hierarchical porous structures of the hydrogel. (a) Schematic of the fabrication method for L-PNIPAm and LSAG. (b) Schematic of the thermally driven water release process for LSAG. (c) Photograph and microstructure of natural loofah sponge and LSAG.

Loofah, a sunlight-dried product from the fully ripened loofah fruit, has an open-pore network interconnected by cellulose fibers (Figure 1). Benefiting from the open-pore structure, a dried loofah sponge has rapid liquid permeation.^43,44^ This inspires our material’s design approach: reconfiguring the overall pore structure of the hydrogel to facilitate faster water transport, thus ultimately overcoming the most limiting drawback in conventional hydrogels. To this end, we developed a loofah-like PNIPAm hydrogel (termed L-PNIPAm) with an interconnected open-pore structure using a poor solvent–water mixture as the polymerization medium. We further functionalized L-PNIPAm with polydopamine (PDA) and poly(sulfobetaine methacrylate) (PSBMA) via an *in situ* polymerization approach, yielding a multifunctional and highly durable loofah-like solar absorber gel (LSAG) for solar water purification (Figure 1). Remarkably, the implementation of LSAG for solar water purification is facile and does not require complicated equipment for operation. The water purification begins with the immersion of LSAG in contaminated water below the LCST, upon which the LSAG swells by absorbing large quantities of water while simultaneously rejecting contaminates. Subsequent exposure to natural sunlight induces solar absorption, which thermally heats the LSAG above the material’s LCST. Following heating, the LSAG undergoes a phase transition and switches from a hydrophilic state to a hydrophobic state. Thus, liquid water is rapidly released from the gel. The expelled liquid water is pure and free from common pollutants, e.g., organic dyes, heavy metals, oils, biological pathogens, and microplastics. The purification mechanism is via adsorption and size rejection, which makes the LSAG highly adaptable to diverse water sources available for potential human consumption. These attributes of LSAG open a new paradigm for solar water production with the potential to meet daily human demand.

## Results and Discussion

### Formation and Characterization of L-PNIPAm

**Figure 2:**
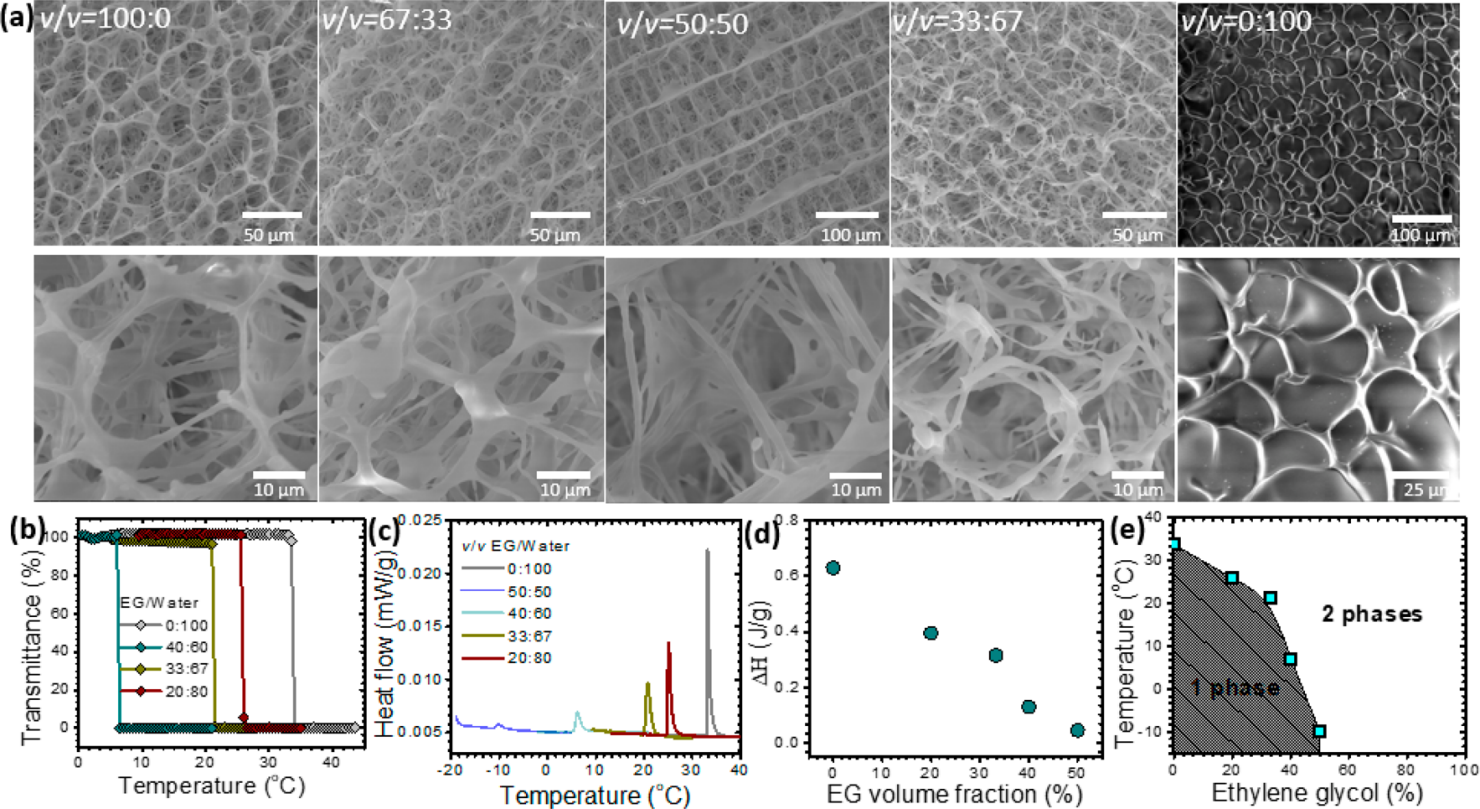
Solvent-mediated morphology and phase transition behavior. (a) SEM images of L-PNIPAm and C-PNIPAm hydrogels synthesized in mixtures of EG and water at room temperature. (b) Temperature-dependent normalized light transmission of C-PNIPAm in EG–water solutions. (c) Phase transition temperatures of C-PNIPAm in EG–water solutions. (d) The enthalpy, Δ*H*, of the phase transition depends on the volume fraction of EG. (e) Phase diagram of C-PNIPAm in EG–water mixtures.

The critical indicator for successfully forming a loofah-like structure is the visible color change during the polymerization process. Generally, hydrogels synthesized in water at ambient temperature are clear and transparent. However, L-PNIPAm hydrogels synthesized in the presence of ethylene glycol (EG) were white and opaque (Figure S1). The difference in appearance implied a difference in network structure. To confirm this difference, the morphology of C-PNIPAm and L-PNIPAm hydrogels was characterized via SEM imaging (Figure 2a). Overall, L-PNIPAm exhibited a hierarchical open-pore structure due to the addition of EG into the polymerization medium. The volume ratio (*v*/*v*) of the EG–water during polymerization strongly influenced the microstructure. For instance, gels polymerized with EG/water ratios of *v*/v = 100/0 and *v*/*v* = 67/33 had a similar overall porous structure, yet the latter exhibited smaller pore sizes and a denser network (Figure 2a). The gel prepared with *v*/*v* = 50/50 had a highly aligned morphology with backbone fibers tightly bridged by short nanofibers. Meanwhile, in a pure water environment (*v*/*v* = 0/100), a regular closed-cell structure was observed. Taken together, the SEM images revealed that EG was critical to facilitating the formation of the open-pore structure, which provides numerous pathways for rapid water transport via capillary flow in response to a temperature change. Finally, we note that room-temperature polymerization is more suitable for forming the open-pore structure regardless of the EG/water ratio. When polymerized at a lower temperature (5 °C), the gel formed using a mixture of *v*/*v* = 33/67 was transparent and exhibited a honeycomb-like structure (Figure S2).

In pure water, C-PNIPAm polymer exhibits an LCST marked by a notable decrease in transmittance (Figure S3). Here, we investigated the influence of EG addition into the aqueous medium on the LCST behavior of C-PNIPAm—that synthesized in pure water (i.e., *v*/*v* = 0/100). As shown in Figure 2b, the addition of EG into aqueous solutions of C-PNIPAm resulted in a lower LCST. This occurred because EG competes with C-PNIPAm for water molecules, which leads to polymer dehydration at a lower temperature. However, at all EG/water volume fractions, a sharp decrease in transmittance was observed. How EG modified the LCST was further investigated via calorimetry. In the DSC thermograms, the endothermic peak, i.e., the LCST calorimetric signature, shifted to a lower value with increasing EG concentration in agreement with transmittance measurements (Figure 2c). A higher volume fraction of EG also resulted in a lower enthalpy of the phase transition (Δ*H*), as determined from the area under the endothermic peaks (Figure 2d).

Figure 2e shows the phase diagram of C-PNIPAm polymer solutions. EG promotes a continuous decrease in the LCST of C-PNIPAm from 34 °C in pure water to −10 °C at 50% EG. Interestingly, in pure EG, C-PNIPAm exhibited an increase in transmittance with increasing temperature from 25 to 85 °C (Figure S4). Hence, C-PNIPAm polymer chains in EG show an upper critical solution temperature (UCST). The appearance of an UCST indicates that EG is a poor solvent for PNIPAm and can trigger its precipitation. To visually observe the EG-induced precipitation of C-PNIPAm, we added EG to a C-PNIPAm–water mixture at ambient temperature. It was observed that the clear solution immediately turned opaque after adding EG (Figure S5). The formation of L-PNIPAm is due to a similar effect: i.e., EG triggers polymer phase separation during polymerization, thus yielding an opaque hydrogel with a unique loofah-like structure (Figure S6a).

We note that the phase separation of C-PNIPAm in mixed solvents has been observed in a series of binary mixtures containing water and an organic solvent such as dimethyl sulfoxide (DMSO),^45,46^ methanol,^47,48^ acetone,^46^ ethanol,^49,50^ and tetrahydrofuran.^51,52^ However, in these cases, both solvents were suitable solvents for C-PNIPAm. Only in a narrow range did their combination result in a poor solvent for C-PNIPAm, in a phenomenon termed the cononsolvency effect. By contrast, here we use a solvent mixture in which one solvent is inherently a poor solvent for PNIPAm, which uniquely distinguishes our process from the cononsolvency phenomenon. Compared to a good solvent (i.e., DMSO) in which gel formation occurs only at high monomer concentration, the poor solvent EG is more suitable for polymerization at low monomer concentration (Figure S6b). Our approach, i.e., the mixed-solvency effect, is facile and could be applied to various hydrogels. Unlike the cononsolvency effect, the precise control of solvent composition is not necessary to achieve an interconnected fibrous structure of hydrogels.^51,53^

### Water Transport within L-PNIPAm

**Figure 3:**
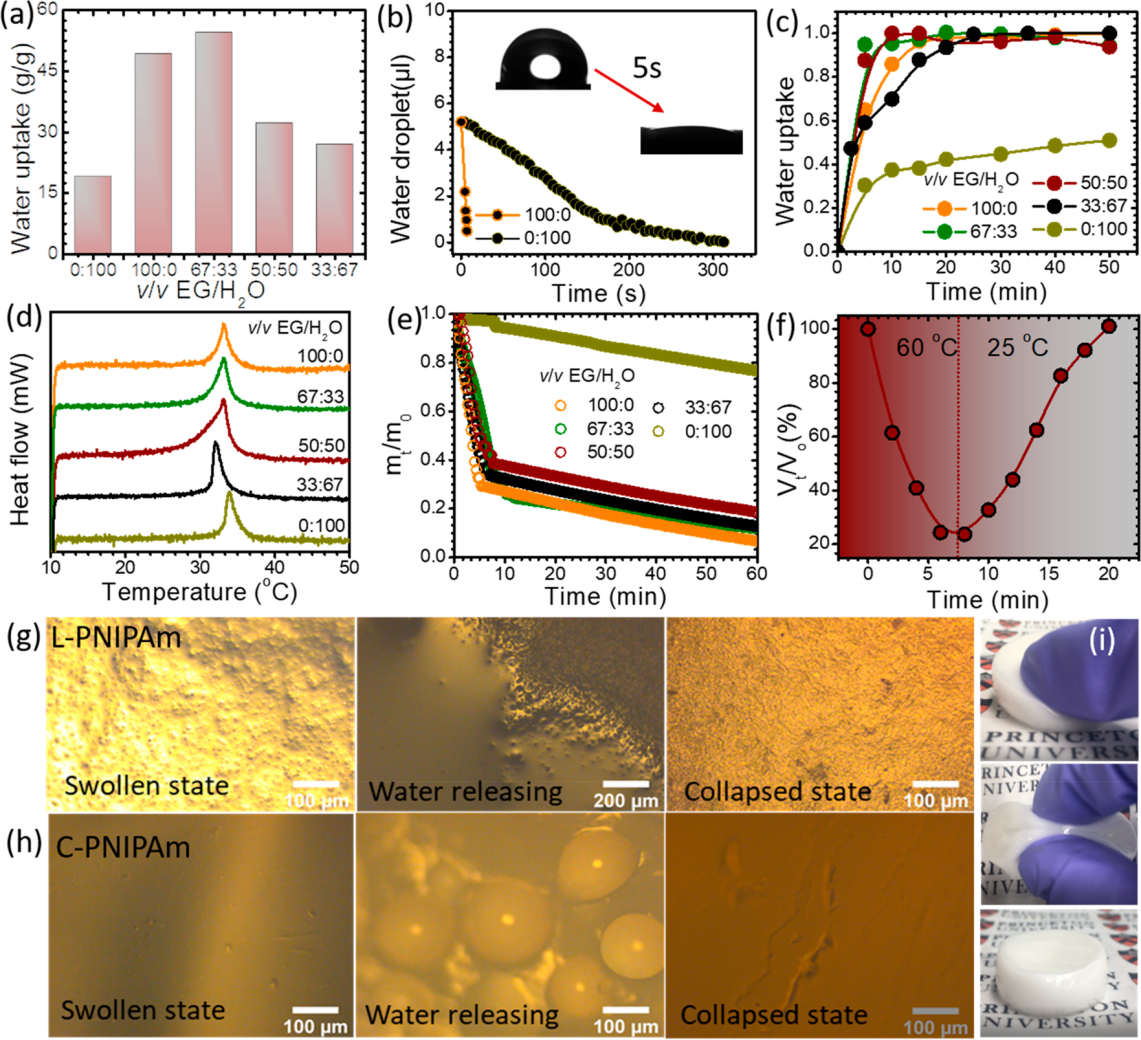
Comparison of thermoresponsive properties. (a) Water uptake of gels polymerized in various EG–water solutions. Wetting behavior (b) and water uptake kinetics (c) of gels with open- and closed-pore structures. (d) DSC thermograms of gels in the swollen state. (e) Water release behavior of gels at 60 °C. (f) Deswelling and swelling of L-PNIPAm upon immersion in water baths at 60 and 25 °C, respectively. Optical microscopy showing the surface change of L-PNIPAm (g) and C-PNIPAm (h) during the water release process. (i) Visual compression and recovery of L-PNIPAm.

Figure 3a compares the water uptake of PNIPAm synthesized in different EG–water mixtures after immersion in water for 12 h. After immersion in water, L-PNIPAm exhibited a marked increase (∼680%) in volume (Figure S7a). In contrast, C-PNIPAm showed no significant volume increase after being immersed in water (Figure S7b). Remarkably, L-PNIPAm (polymerized in *v*/*v* = 67/33) exhibited water uptake over 3 times higher than that of C-PNIPAm (Figure 3a). The enhanced water uptake is attributed to the interconnected open pores in the gel, consistent with SEM images (Figure 2a). The effect of pore structure on water uptake was further investigated by recording the dynamic wetting behavior of a water droplet using lyophilized gels. As shown in Figure 3b, a water droplet atop the surface of L-PNIPAm penetrated the gel within ∼7 s, while for the C-PNIPAm, it took ∼300 s (Movie S1). This difference in surface adsorption dramatically affected the water uptake kinetics of gel membranes, as shown in Figure 3c. Gels with an open-pore structure exhibited faster water uptake kinetics. This attribute, combined with an overall higher level of water uptake, makes L-PNIPAm a particularly attractive thermoresponsive material for water treatment and collection technologies.

An accessible LCST is critical for using L-PNIPAm as a thermoresponsive material. Interestingly, L-PNIPAm exhibits an LCST (∼34 °C) similar to that of C-PNIPAm, as measured by DSC (Figure 3d). Above the LCST, it was anticipated that L-PNIPAm would expel absorbed water similarly to C-PNIPAm. We tested the water release kinetics of L-PNIPAm by placing it at 60 °C and a relative humidity of ∼50%. As shown in Figure 3e and Figure S8, the water release of L-PNIPAm exhibited two regimes distinguishable by a sharp change in release rate. At short times (<5 min), the water release rate of L-PNIPAm was substantially higher, while at longer times (>5 min), the rate of water release approached that of C-PNIPAm. In particular, L-PNIPAm released ∼70% of its absorbed water within 5 min. In contrast, C-PNIPAm released only ∼3% of its absorbed water over the same time (Figure 3e and Figure S8). This difference is a direct result of morphological differences. When C-PNIPAm is heated above its LCST, a thick and dense skin layer forms at the surface, as visualized by the appearance of water-containing bubbles at the surface (Figure S9). The dense layer prevents the rapid release of free water, leading to a relatively low initial deswelling rate.^39^ In contrast, for L-PNIPAm, no dense skin layer was formed due to the unique loofah-like structure (Figure S9). We also note that the high water release rate of the L-PNIPAm gel was maintained in a hot water (60 °C) environment, and the collapsed gel could quickly reswell upon transferring it into cold water (Figure 3f).

The difference in water release kinetics between L-PNIPAm and C-PNIPAm was also visually observed by monitoring water release from the gels’ surface via optical microscopy. Figure 3g shows water release from the surface of L-PNIPAm. In the swollen state, open pores were visible at the surface. Therefore, upon heating above the LCST, the surface of L-PNIPAm was “flooded” with a layer of water due to release from numerous surface pores. A different water release mechanism was observed for C-PNIPAm, as shown in Figure 3h. First, we note that open pores were not visible at the surface of C-PNIPAm. After heating above the LCST, small water droplets appeared at the surface and grew into larger droplets. Finally, the difference in morphology also manifests dramatic changes in mechanical properties. As expected, C-PNIPAm was a brittle material and could not sustain compression (Figure S10). In contrast, L-PNIPAm with two different monomer concentrations (5 and 20 wt %) could sustain compression and recover its original shape upon stress removal, as illustrated in Figure 3i and Figure S10. Even though it is difficult to quantify the cross-link density of the gel, similar open-pore structures and rapid-response behaviors were observed for two different gels synthesized with different monomer concentrations, while maintaining the same Bis cross-linker concentration. The observation suggests that the rapid release property of L-PNIPAm is due to the open-pore structure.

### Water Transport and Antifouling Characterization of LSAG

**Figure 4:**
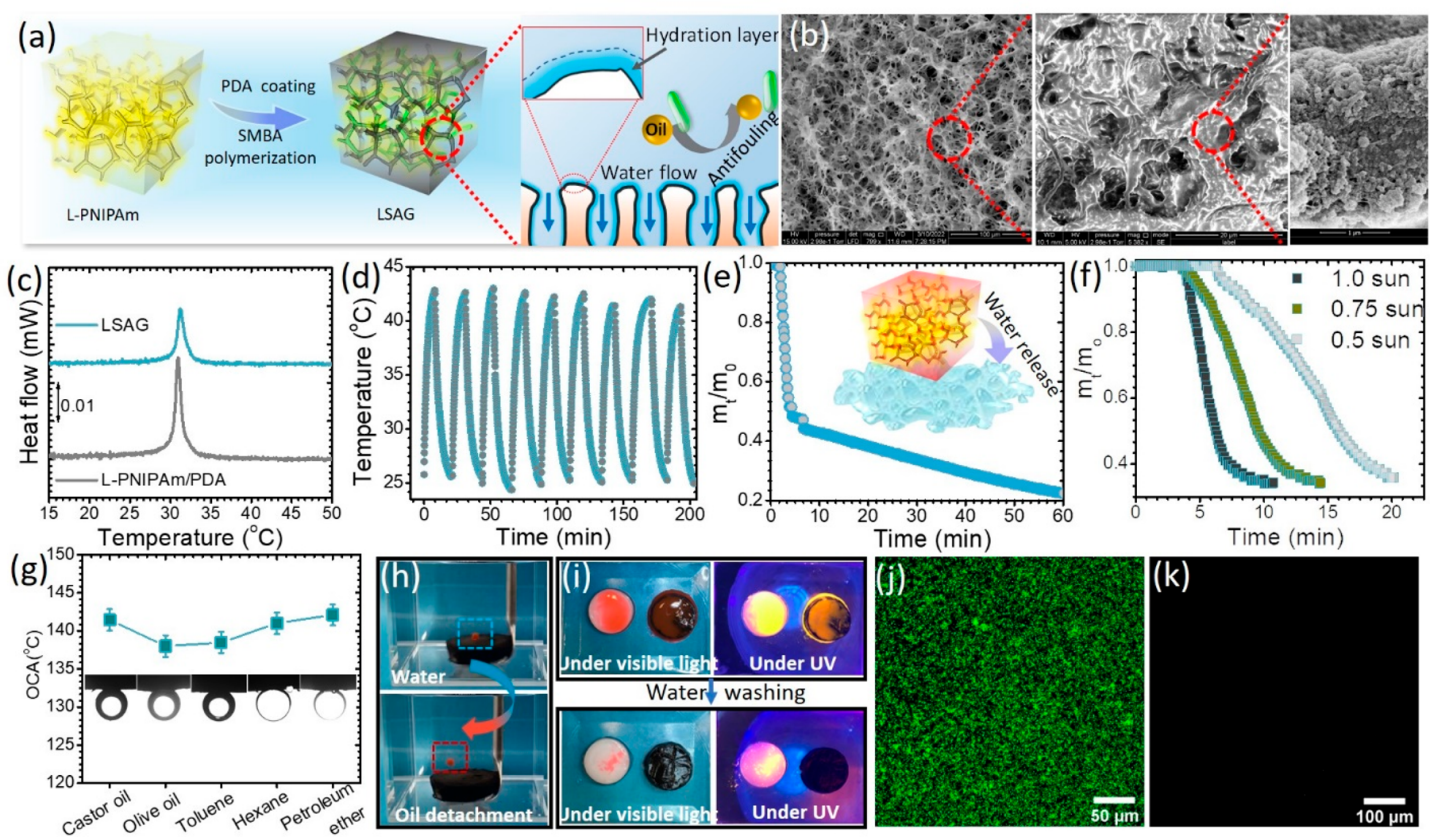
Design of gels with photothermal and antifouling capabilities. (a) Schematic representation of the LSAG formation and its antifouling behavior. (b) SEM images with different resolutions of LSAG. (c) DSC thermograms of the gels before and after the PSBMA modification. (d) Results of a nine-cycle photothermal test on LSAG under 1 sun illumination. (e) Mass change of LSAG upon heating at 60 °C. (f) Mass change of LSAG over time under simulated sunlight illumination with various intensities. (g) Underwater oil contact angle (OCA) of LSAG with different oils. (h) Photos of LSAG fouled by a droplet of Nile red labeled olive oil after being immersed in water. (i) Photos, taken under visible and UV light, of the L-PNIPAm gel (opaque) and the LSAG (black) contaminated by Nile red labeled olive oil in air before and after being washed by water. Fluorescence microscopy images of *E. coli* adsorption atop a (j) glass slide and (k) LSAG, respectively.

The functionalization of L-PNIPAm with PDA and PSMBA resulted in an LSAG membrane with a dark complexion (Figure S11) as well as antifouling capabilities (Figure 4a). SEM images reveal that the addition of PDA and PSMBA to L-PNIPAm did not alter the interconnected open-pore structure (see Figure 4b). Higher magnification revealed that PDA was deposited atop the network structure in the form of nanoparticles (see Figure 4b). Energy-dispersive X-ray (EDX) elemental mappings showed the existence of S K-edge elements throughout the LSAG (see Figure S11), confirming the successful formation of the PSBMA network within the gel. The LCST of LSAG was ∼31 °C, as identified by the endothermic peak in the DSC thermogram (see Figure 4c). The slightly lower LCST is critical to enable low-energy water release. Indeed, upon exposure to simulated sunlight of 1 kW m^–2^ (1 sun), the core temperature of swollen LSAG increased with time and eventually reached ∼43 °C within 4 min (see Figure S12a). The fast photothermal response indicates shorter times and lower energy are required to reach the LCST and trigger water production. Critical to the performance of the technology is thermal cyclability. The LSAG showed no evidence of deterioration of the photothermal response, e.g., response rate and surface temperature (∼41.5 °C), with an increasing number of cycles (see Figure 4d).

When heated above the LCST to ∼60 °C, a swollen LSAG rapidly released the absorbed water similarly to L-PNIPAm, as shown in Figure 4e. Remarkably, the LSAG can rapidly expel water when exposed to simulated sunlight with various intensities (see Figure 4f and Figure S12b). Under 1 sun irradiation, an ∼4 min induction time was observed prior to water release. Subsequently, a nearly linear decrease in mass loss (water release) was observed with time, eventually reaching ∼70% of stored water release within ∼10 min. As a result, the water collection rate reached 26.88 kg m^–2^ h^–1^, i.e., ∼4 times higher than that of the previously reported SAG (7.18 kg m^–2^ h^–1^).^22^ This difference makes LSAG a water purification technology with the potential to meet daily water demand. Considering the variability in solar intensity, the effect of illumination intensity on the LSAG’s deswelling behavior was also investigated by exposing it to lower-intensity sunlight, including 0.75 kW m^–2^ (0.75 sun) and 0.5 kW m^–2^ (0.5 sun). Under these conditions, ∼15 and 20 min were needed, respectively, to release ∼70% of the stored water. The deswelling performance indicates that LSAG can operate under reasonably low intensity illumination.

The underwater oil contact angles (OCA) of LSAG were measured after being immersed in water (see Figure 4g). The LSAG exhibited an OCA of ∼140° for a variety of oils, thus exhibiting the characteristic of underwater superoleophobicity. The antioil fouling performance of LSAG was tested using a dye-labeled oil. In an air environment, the oil droplet readily spreads atop the surface of LSAG. In contrast, as shown in Figure 4h and Movie S2, when placed in an aqueous environment, the flat oil layer spontaneously transitioned into a single droplet and completely detached from the surface within 2 s. The fast and complete oil detachment in water indicates oil antifouling and self-cleaning capabilities. This was further confirmed by fouling the surface of LSAG, as shown in Figure 4i. The LSAG surface could be thoroughly rejuvenated by washing in water, as evidenced by the absence of Nile red luminescence under UV light. In comparison, L-PNIPAm, which does not include the zwitterionic functionality, retained residual oil atop its surface.

Building on the oil antifouling capabilities of LSAG, its bio antifouling performance was tested using *Escherichia coli* (*E. coli*) as a model bacterium and compared it with that of silanized glass as well as an L-PNIPAm/PDA gel without PSBMA as control samples, as shown in Figure 4j,k and Figure S13. As revealed by confocal laser scanning microscopy, significant *E. coli* adhered to the glass surface and the L-PNIPAm/PDA gel (∼20% surface coverage), while its adhesion was negligible atop LSAG (∼0.08% surface coverage). The antifouling properties of LSAG can be explained as follows: the binding of charged units of zwitterionic PSBMA with water molecules generates a hydration water layer at the interface, which helps to prevent containment adhesion as illustrated in Figure 4a.^54−57^ These results further confirm our design principle of an antifouling hydrogel by introducing zwitterionic PSBMA into the L-PNIPAm.

### Water Purification Performance of LSAG

The low energy requirement and high water collection rate, combined with the demonstrated antifouling properties of LSAG, suggest a materials platform suitable for sustainable wastewater purification. We tested LSAG’s water decontamination capability in multiple model wastewater feedstocks containing small-molecule dyes, heavy metals, oil, and microplastic particles. The procedure for water purification was as follows: (1) LSAG was immersed into the contaminated water resource to swell, (2) LSAG was removed and irradiated by 1 sun, and (3) the collected water was analyzed for purity.

**Figure 5:**
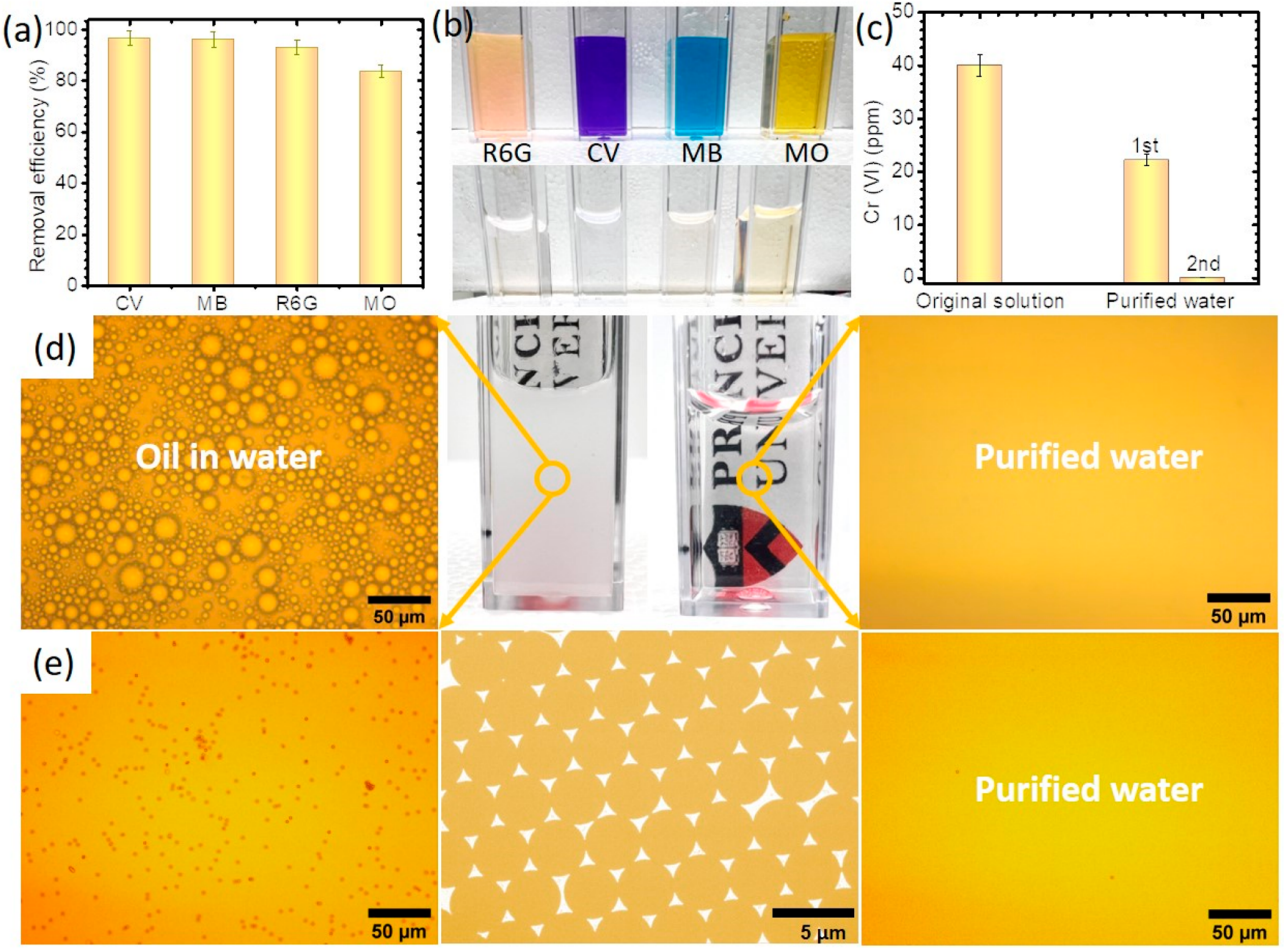
Water purification performance of LSAG. (a) Water purification performance of LSAG toward various dye-contaminated water samples. (b) Photographs of the contaminated water and the generated water treatment by LSAG. (c) Concentration of Cr(VI) in water purified by LSAG. (d) Digital and microscope images of an SDS-stabilized olive oil-in-water emulsion and the purified water by LSAG. (e) Optical microscope and TEM images (in the middle) of a microplastic particle solution before and after treatment by LSAG.

First, positively charged organic dyes (Rhodamine 6G (R6G), methyl blue (MB), and crystal violet (CV)), and negatively charged methyl orange (MO) were selected as representative model pollutants to evaluate the LSAG’s water purification property. For R6G, MB, CV, and MO, LSAG showed high removal efficiencies of ∼94%, ∼96%, ∼97.0%, and ∼84%, respectively, after one treatment cycle (Figure 5a and Figure S14). The high purity of the produced water can also be observed from the color difference between contaminated and purified water, as depicted in Figure 5b. The LSAG can also be used to remove a heavy metal, e.g., Cr(VI), as shown in Figure 5c. After a two-cycle treatment, the purified water contained less than 0.07 ppm of Cr(VI), which is below the EPA allowable limits for drinking water. To meet the needs of practical applications, the cycling stability of the LSAG must be considered, which can be easily achieved by washing it with ethanol.^58,59^ There is no reduction in the removal efficiency of CV from contaminated water and the water production rate after 10 adsorption–desorption cycles (Figure S15). Oil pollution is one of the most serious threats to water sources.^44,60^ Here, we demonstrate LSAG’s ability to generate pure water from an oil-in-water emulsion (see Figure 5d and Figure S16). Purified water from the oil-contaminated water was absent of visible oil droplets. The excellent decontamination capability of LSAG toward dyes and heavy metals is ascribed to the PDA’s superior adsorption property, which has a high density of amine and catechol groups for contaminant removal.^61^ The LSAG’s ability to create clean water from an oil-in-water emulsion is attributed to the existence of zwitterionic PSBMA. Its excellent superhydrophilicity and underwater superoleophobicity allows the LSAG to absorb water but repel oil droplets,^44,62^ which is consistent with the antifouling testing.

Finally, we show that LSAG can also be used to remove microplastics from contaminated water. Microplastics are synthetic hydrocarbon-based particles that have become an emerging environmental pollutant threatening public health.^63,64^ According to the World Health Organization (WHO), microplastics are ubiquitous and have been detected in oceans, lakes, rivers, tap water, and bottled water.^65^ To test the microplastic filtration capability of LSAG, colloidal solutions of polystyrene (PS) nanoparticles (0.1%, ∼3.2 μm) and irregular silica particles (1%, ∼50 μm) were used as models of environmentally persistent microplastic-contaminated water. The original microplastic suspensions are opaque, and the well-dispersed particles can be seen from the microscopic photographs (Figure S17). In contrast, the LSAG-purified water from these two suspensions is clear and transparent. From the microscopic graphs, no noticeable silica particles could be clearly observed in the purified water. For the smaller PS nanoparticles, the LSAG shows good filtration properties, as most of the PS particles were filtered out by LSAG after treatment (Figure 5e). It should be noted that during the swelling of LSAG in the PS nanoparticle suspension, the final swelling volume of the gel, i.e., the pore size of the network, needs to be controlled to achieve successful filtration of small microplastics.

## Conclusion

In this study, we developed L-PNIPAm with a unique loofah-like structure using the mixed-solvency effect, which was further modified with PDA and PSMBA, yielding a multifunctional water purification system: i.e., LSAG. The loofah structural feature enabled the L-PNIPAm to have a 3-fold enhancement in swelling ratio, ultrafast water transport, as well as improved mechanical properties when compared to C-PNIPAm. Specifically, only ∼5 min was needed to release ∼70% of the water from L-PNIPAm, demonstrating the loofah structure’s role as a breakthrough to overcome the inherent slow response rate. The LSAG has the potential to purify water from various contaminated sources powered by natural sunlight. The antifouling and quick release properties of LSAG make it adaptable for operation in complex, practical environments. These merits substantiate LSAG’s potential to provide facile and affordable access to clean water in a sustainable, low-energy way to the world’s population.

## Data Availability

The data that support the findings of this study are available from the corresponding author upon request. All other data needed to evaluate the conclusions in this study are provided in either the manuscript or the Supporting Information.

## Supporting Information Available

Figures S1 to S17 Movies S1 to S2 The Supporting Information is available free of charge at https://pubs.acs.org/doi/10.1021/acscentsci.2c01245.

Experimental section and additional figures as described in the text (PDF)
Water diffusion test of conventional PNIPAm hydrogel and the loofah-like PNIPAm hydrogel (MP4)
Oil antifouling property of LSAG (MP4)
