# Supplementary material for: Quick-Release Antifouling Hydrogels for Solar-Driven Water Purification
Source: ACS Cent Sci. 2023 Feb 8;9(2):177–85. doi: 10.1021/acscentsci.2c01245 (PMC9951281; doi:10.1021/acscentsci.2c01245)
Supplement: Supplementary file 1 — oc2c01245_si_001.pdf [file oc2c01245_si_001.pdf]

Supporting Information for

Quick Release Anti-Fouling Hydrogels for Solar-Driven Water Purification

Xiaohui Xu[1], Néhémie Guillomaitre[1,2], Kofi S.S. Christie[4,5], R. Kōnane Bay[1], Navid Bizmark[1,3], Sujit S. Datta[1], Zhiyong Jason Ren[4,5], Rodney D. Priestley[1,3*]

[1]Department of Chemical and Biological Engineering

[2]Mechanical and Aerospace Engineering Department

[3]Princeton Materials Institute

[4]Department of Civil and Environmental Engineering

[5]Andlinger Center for Energy and the Environment,

Princeton University, Princeton, New Jersey 08540, United States

*Corresponding author email: rpriestl@princeton.edu

This PDF file includes the following:

Experimental Section

Supplementary Figures S1 to S17

Captions for Movies S1 and S2

Supporting References

Table of Contents

Experimental Section S4-7

Materials

Preparation of open-cell structure L-PNIPAm gels

Preparation of photothermal antifouling gels

Measurement of phase transition temperatures

Measurement of water release performance

Measurement of water purification performance

Measurement of anti-biofouling

Materials Characterizations

Supplementary Figures S7-23

Fig. S1. (a) The chemical structures of the monomers and crosslinkers used in the polymerization process. (b) Optical photos of PNIPAm gels polymerized in EG-water mixtures with different volume fraction ratios.

Fig. S2. (a) SEM images of PNIPAm gels polymerized at 5 °C with an EG–water ratio of 33:67. (b) The water release rate of gels (polymerized at 5 °C and room temperature) upon heating at 60 °C.

Fig. S3. A reversible coil-to-globule transition of PNIPAm polymer in pure water and pure EG.

Fig.S4. UCST behavior of PNIPAm in pure EG.

Fig. S5. Phase separation of a PNIPAm aqueous solution upon the addition of EG.

Fig. S6. (a) The polymerization process of L-PNIPAm gel (*v*/*v*=67:33). (b) Polymerization of NIPAm in DMSO at room temperature and 60 °C, respectively.

Fig. S7. (a) L-PNIPAm and (b) C-PNIPAm (made in pure water) before and after swelling in water.

Fig. S8. (a) Time dependence of the deswelling behavior of PNIPAm at 60 °C. Rate analysis of the deswelling properties of PNIPAm synthesized in (b) water and (c) EG. (d) The complete water release profile of L-PNIPAm (polymerized in EG) at 60 °C.

Fig. S9. Surface change of (a) C-PNIPAm and (b) L-PNIPAm upon heating above the LCST.

Fig. S10. (a) C-PNIPAm and (b) L-PNIPAm under compression. (c, d) Mechanical test of PNIPAm with different EG-water ratios before swelling in water. (e) Photographs of swollen L-PNIPAm under a compression–decompression test cycle at a compressive strain of 50 %; and (f) the corresponding reversible compressive stress-strain curve.

Fig. S11. Photographs and EDX mapping results of LSAG.

Fig. S12. Photothermal effect of LSAG under one sun irradiation. (a) The internal temperature change of a swollen LSAG. (b) Photographs of a swollen LSAG before and after one sun irradiation.

Fig. S13. (a) Fluorescence microscopy images of *E. coli* adsorption atop an L-PNIPAm/PDA gel surface with an average coverage of 20.8%. (b) Average coverage of *E. coli* atop a glass slide and LSAG after 24 hr of culture.

Fig. S14. UV-Vis measurement of the original dye solutions (CV, MB, R6G, MO) and purified water *via* LSAG.

Fig. S15. Recycle stability of the LSAG for CV removal. (a, b) UV-Vis spectrum of the produced water from LSAG with different cycles. (c) Removal efficiency of LSAG. (d) The mass change during the recycling of LSAG; the shaded regions indicate that the LSAG was under 1 sun irradiation for 10 min. (e) The water production rate of LSAG during the decontamination process.

Fig. S16. Original SDS-stabilized olive oil-in-water emulsion and the purified water *via* LSAG.

Fig. S17. (a) Purification process of water containing microplastic particles. (b) PS circular particles with a diameter of 3.2 μm. (c) Irregular silica particles with a diameter of 50 μm.

Movie S1. The water diffusion test of conventional PNIPAm hydrogel and the loofah-like PNIPAm hydrogel.

Movie S2. The anti-oil fouling property of LSAG.

Supporting References S23

Experimental Section

Materials: Dopamine hydrochloride, Tris-base, *N*-isopropylacrylamide (NIPAm), *N*,*N*′-methylenebisacrylamide (Bis), potassium persulfate (KPS, $K_2S_2O_8$), Irgacure 2959, *N*,*N*,*N*′,*N*′-tetramethyl-ethylenediamine (TEMED), ethylene glycol (EG), polyethylene (glycol) diacrylate (PEGDA, *Mn* 700), and *N*-(3-sulfopropyl)-*N*-(methacryloxyethyl)-*N*,*N*-dimethylammonium betaine (SBMA, 97%) were obtained from Sigma-Aldrich and used without further purification.

Preparation of open-cell structure L-PNIPAm gels: PNIPAm hydrogels were synthesized by free radical polymerization in different mixtures of EG and water. Specifically, 230 mg of NIPAM, 8 mg of Bis, and 5 mg of KPS were dissolved in 4 mL of mixed EG and water under magnetic stirring. The above mixture was combined with 10 μL TEMED and sealed at room temperature overnight to form opaque PNIPAm gels. The chemical structures and the appearance of the obtained hydrogels can be found in **Fig. S1**. The PNIPAm hydrogels were soaked in DI water for 24 hours until the unreacted precursors and EG were removed. To investigate the effect of EG on the thermoresponsive behavior, PNIPAm linear chains were also fabricated in DI water using the same procedure described above but without the addition of the Bis crosslinker. To explore the effect of mole fraction on pore structure and improve the mechanical properties, 20 wt.% NIPAm prepolymer in EG were also used to fabricate gels for practical applications. In addition to covalent crosslinking with Bis, additional chain entanglements (physical crosslinking) due to an increase in monomer concentration can increase the crosslinking density of the gel at higher monomer content, thus improving the mechanical properties of the gel.

Preparation of photothermal antifouling gels: The obtained PNIPAm gel with 20 wt.% NIPAm was wetted with a dopamine-Tris buffer solution (20 mg/mL) for 6 h. This procedure was repeated four times until a dark gel was formed. To maintain the original loofah-like morphology, the polymerization of SBMA was performed according to a previously reported method.[1] Briefly, the dark gel was immersed in an SBMA solution (2 M) containing Irgacure 2959 as the photoinitiator and 0.2 % PEGDA as the crosslinker (the chemical structures are shown in **Fig. S1**). Subsequently, the gel was frozen at -20 °C overnight and illuminated with UV light for 4 h to induce the polymerization of PSBMA within the gel network. To avoid gel shrinking due to heat generated by the UV lamp, dry ice was put inside the UV chamber to cool the environment during polymerization.

Measurement of phase transition temperature: Before each measurement, PNIPAm was dissolved in pure water and EG-water mixtures at a polymer concentration of 10 g L^{-1}. In pure EG, the PNIPAm concentration was about 3 g L^{-1}. The phase transition of PNIPAm in different mixtures of EG and water was determined by transmittance measurements using dynamic light scattering (DLS, Anton Paar Litesizer 500) and differential scanning calorimetry (TA DSC-8500). For DSC measurements, 30 μL samples were loaded in aluminum cells and sealed hermetically. The thermal analysis was performed in a temperature range from -20 °C to 40 °C with a heating rate of 1 °C min^{-1} in a dry N_2 atmosphere (with a flow rate of 50 mL min^{-1}). The enthalpy value of the phase transition was determined from the area under the endothermic peak normalized by the mass of the solution in units of J g^{-1}. For the phase transition measurement of crosslinked gels, all samples were first washed with DI water to remove the EG, then the measurements were carried out from 10 to 40 °C.

Measurement of water release performance: The water release behavior of PNIPAm was performed by placing the samples in a controlled environment at 60 °C and 30 % relative humidity. The mass change of the gels was recorded every 5 s by a digital balance. For the photothermal systems, the swollen gels were exposed to simulated sunlight using a 300 W xenon arc lamp with light intensities of 0.5, 0.75, and 1 sun at ambient temperature of 20 °C. For the thermal cyclability measurements, the air-dried LSAG was irradiated by 1 sun. Temperature variation with the gel was measured using a thermometer at select time intervals.

Measurement of water purification performance: The water purification capability of LSAG was tested using water containing one of four model contaminants: hexavalent chromium [Cr(VI)], emulsified olive oil, molecular dyes, and microplastics. Solutions of 40 ppm Cr(VI) were used to analyze the removal of heavy metals from contaminated groundwater. A two-cycle treatment was used to remove Cr(VI): For cycle one, an LSAG was placed in a solution containing Cr(VI); subsequently, the adsorbed water was released under sunlight. For cycle two, a new LSAG was submerged into the water released from the first cycle; thereafter, the adsorbed water was removed from the second LSAG. To explore the potential for oil wastewater treatment, an olive oil-in-water emulsion was prepared by mixing 2 ml olive oil with a 100 mL SDS water solution (1 mg L^{-1}) and stirred at 1000 rpm overnight. Four aqueous solutions of representative dyes (Rhodamine 6G, methyl blue, methyl orange, and crystal violet), each at a concentration of 10 mg L^{-1}, were prepared to analyze the dye removal capacity of LSAG. The water purification process was carried out by

immersing dry LSAG into the prepared mixtures. Subsequently, the swollen LSAG was removed from the solution and solar-heated above the LCST to trigger water release. To investigate the microplastic removal property of LSAG, regular polystyrene (PS) particles and irregular silica particles with varied sizes were dispersed into water, forming opaque suspensions. To ensure the filtration efficiency of LSAG for small PS particles (3.2 μm), the swollen volume, i.e., the pore size of the network, must be controlled by encasing LSAG in a rigid mold with a diameter of 15 mm (**Fig. S17a**). The mold was used to prevent the over-swelling of LSAG. Then the dry LSAG in the mold was immersed into a suspension of PS nanoparticles overnight. For the filtration of large silica nanoparticles, the dry LSAG was immersed into 10 mL silica suspension (1 wt.%) in a glass vial and stirred until swelling equilibrium was achieved. In the end, the swollen LSAG was allowed to release water *via* heating above the LCST. For the filtration of PS particles (3.2 μm), we use a rigid plastic mold with a diameter of 15 mm to surround the gel. Then the dry LSAG in the mode was immersed into a suspension of PS nanoparticles. During water absorption, the volume of LSAG was restricted during the swelling process, which was shown to be beneficial for the filtration of the microplastic particles.

Measurement of anti-biofouling: Before each assay, we prepared an overnight culture of *E. coli* (W3110) that constitutively expresses green fluorescent protein (GFP) throughout the cytoplasm by shaking at 150 rpm at 37 °C in Luria-Broth (LB). We also tested a strain containing a deletion of the flagellar regulatory gene flhDC, which does not assemble flagella. The 300 μL cells were harvested from the overnight culture at the stationary phase ~ 10^9 cells mL^{-1}. A 24 h quiescent adhesion assay was used to determine the bacterial adhesion to the hydrogels, which were compared to circular salinized cover glass. Specifically, the prepared hydrogels were first swollen in a 1x PBS and then placed in a 6-well plate (Cellvis) containing 6 mL of LB and 300 μL of a bacteria suspension. The samples were incubated at 30 °C for 24 h without shaking. After incubation, samples were dipped into 1x PBS to remove loosely adhered bacteria. To measure the area coverage of the samples, we use a 20x lens and Nikon A1R inverted laser scanning confocal microscope to capture fluorescence images of 15 random locations (area of the location). The images were binarized then analyzed using the ImageJ "Analyze particle" function to determine the area coverage on the samples.

Materials Characterizations: The morphologies of the as-prepared gels were characterized by scanning electron microscopy (Quanta 200 FE-ESEM and Verios 460 XHR SEM). The PS

particles were imaged using a Thermo Fisher Scientific Talos F200X TEM. Underwater oil contact angles were measured on an OCA20 machine (Data Physics). The absorbance spectra of the wet samples were measured on a UV-Vis-NIR spectrometer (Agilent Technologies, Cary 5000).

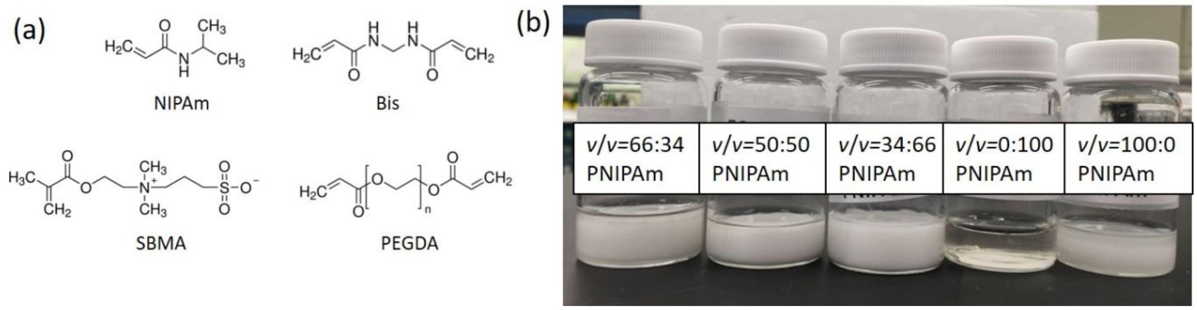

Fig. S1. (a) The chemical structures of the monomers and crosslinkers used in the polymerization process. (b) Optical photos of PNIPAm gels polymerized in EG-water mixtures with different volume fraction ratios.

(a) (b)
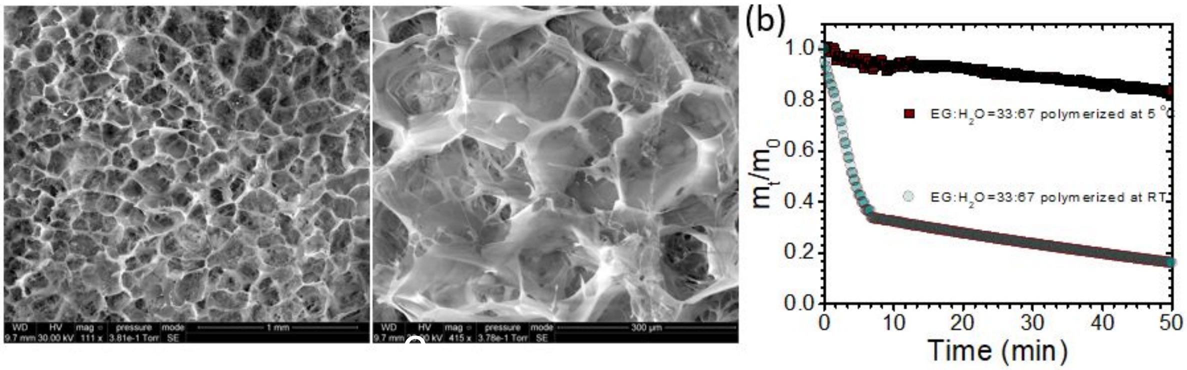

Fig. S2. (a) SEM images of PNIPAm gels polymerized at 5 °C with an EG–water ratio of 33:67. (b) The water release rate of gels (polymerized at 5 °C and room temperature) upon heating at 60 °C.

Fig. S2a presents the SEM images of PNIPAm polymerized at 5 °C. As illustrated, freeze-dried PNIPAm exhibits a porous structure with connected polymeric walls, forming a honeycomb structure. In these pores, some crosslinked fibers can be seen. This is due to the coexistence of water and EG and the low polymerization temperature. **Fig. S2b** shows the difference in water release rate for gels made using the EG-water ratio but at a different temperature. The gel made at room temperature has a higher water release rate than that made at 5 °C, which further suggests their difference in network structure.

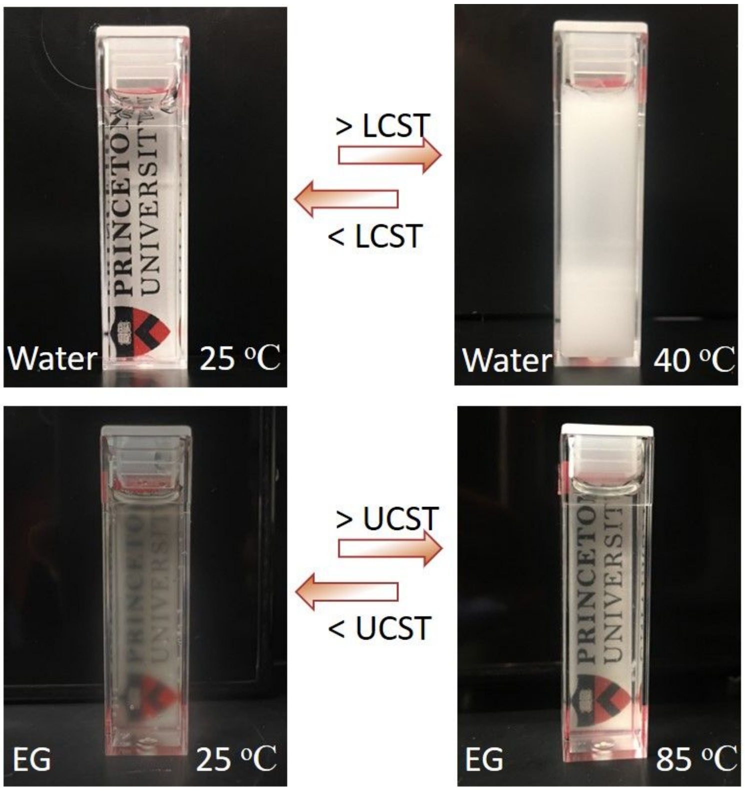

Fig. S3. A reversible coil-to-globule transition of PNIPAm polymer in pure water and pure EG.

PNIPAM chains exist in the coil state in pure water at room temperature and collapse into globules above the LCST. While in pure EG solvent, the PNIPAm solution is clear at 90 °C and cloudy at room temperature, indicating its UCST behavior.

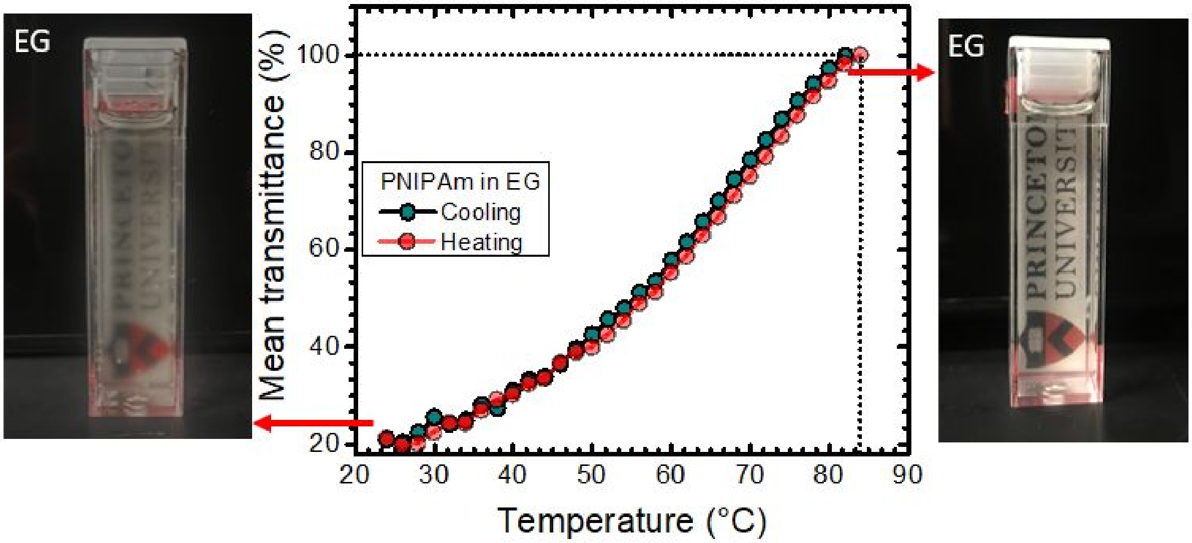

Fig. S4. UCST behavior of PNIPAm in pure EG.

An interesting observation in **Fig. S4** is that PNIPAm in pure EG displayed an increase in transmittance from 20 % to 100 % when the temperature was increased from 25 to 85 °C. This temperature-enhanced solubility suggests that the PNIPAm-EG system has an upper critical solution temperature (UCST). The appearance of a UCST indicates that EG is a poor solvent for PNIPAm.

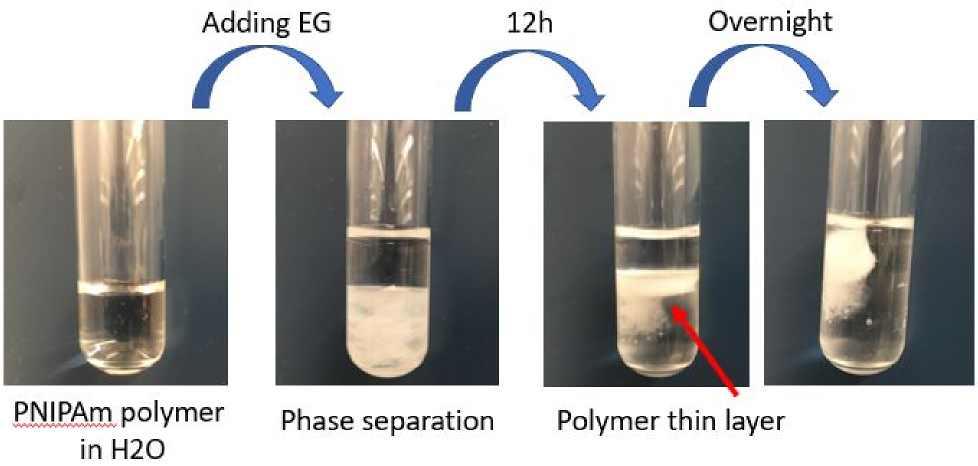

Fig. S5. Phase separation of a PNIPAm aqueous solution upon the addition of EG.

We added EG into a PNIPAm-water mixture at ambient temperature to visually observe the EG-induced precipitation. In pure water, the PNIPAm polymer chains are saturated with water molecules, forming a transparent solution; while right after the addition of EG, the clear solution immediately turned opaque, indicating the coil-to-globule transition. This phenomenon is because EG is a bad solvent for PNIPAm and a good solvent for water. It, therefore, competes with the PNIPAm for water molecules. The result is that PNIPAm dehydrates and precipitates out of the water phase *via* hydrophobic aggregation.

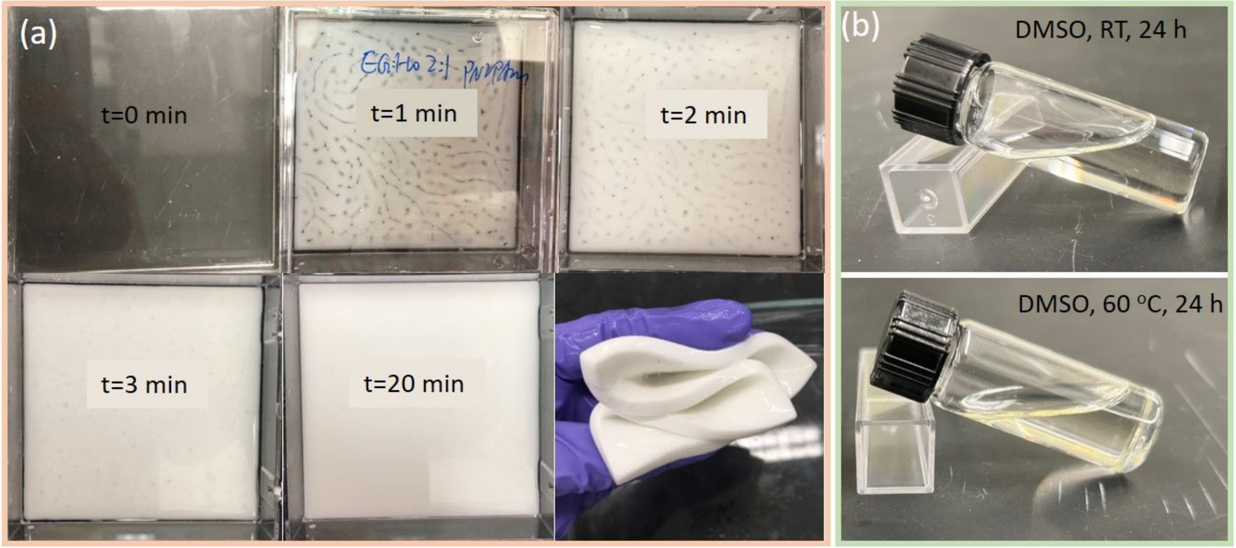

Fig. S6. (a) The polymerization process of L-PNIPAm gel (*v*/*v* = 67:33). (b) Polymerization of NIPAm in DMSO at room temperature and 60 °C, respectively.

The formation of L-PNIPAm in mixed solvents proceeds by the following process: the monomer undergoes chain propagation and crosslinking and subsequent solvent–polymer phase separation, thus yielding an opaque hydrogel with a unique loofah-like structure (**Fig.S6a**). The gel formation process was also observed in pure EG. However, the same monomer concentration could not form PNIPAm in pure DMSO at room temperature or 60 °C for 24 hours. Only at high monomer concentration (i.e., 2 g/mL) PNIPAm hydrogel can be obtained in DMSO. This indicates that the polymerization of NIPAm in a good solvent such as DMSO is concentration-dependent.

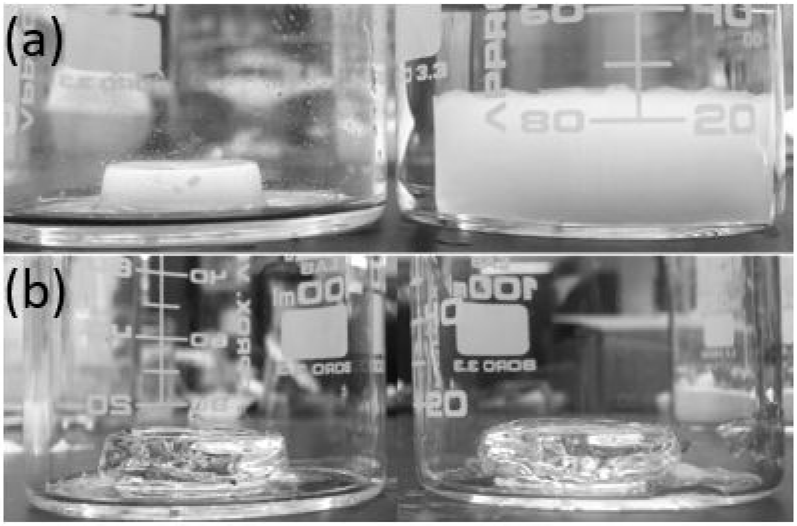

Fig. S7. (a) L-PNIPAm and (b) C-PNIPAm (made in pure water) before and after swelling in water.

Fig. S7 compares the water uptake of L-PNIPAm and C-PNIPAm obtained upon immersing the gels in water overnight. After water absorption, opaque L-PNIPAm exhibited a marked change in volume (from 1.885 cm^3 to 12.83 cm^3) and transformed into a highly expanded network. In contrast, the volume of C-PNIPAm did not significantly change. The difference in volume change between the two gels is because of the difference in their network microstructures (open pore vs. closed pore).

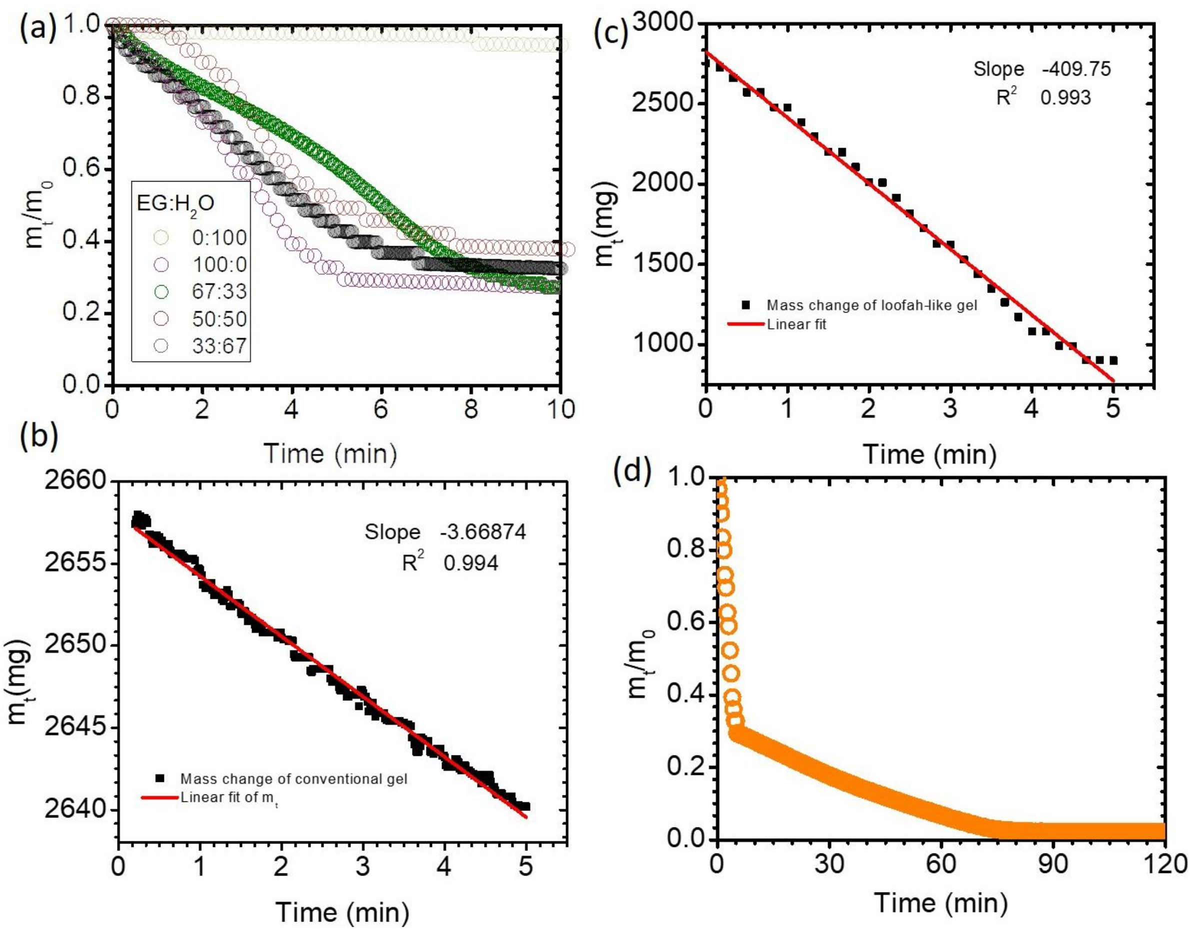

Fig. S8. (a) Time dependence of the deswelling behavior of PNIPAm at 60 °C. Rate analysis of the deswelling properties of PNIPAm synthesized in (b) water and (c) EG. (d) The complete water release profile of L-PNIPAm (polymerized in EG) at 60 °C.

L-PNIPAm synthesized in pure EG can release ~ 70 % of its stored water within 5 min. In sharp contrast, only ~ 3 % of the stored water was released in C-PNIPAm within the same time frame due to the formation of a skin layer that blocked the water flow from the gel. More importantly, for L-PNIPAm, the initial rapid water release process is independent of the EG fraction used during polymerization. In contrast, the water release profile of gels made via the cononsolvency effect strongly depends on the solvent ratio during polymerization.

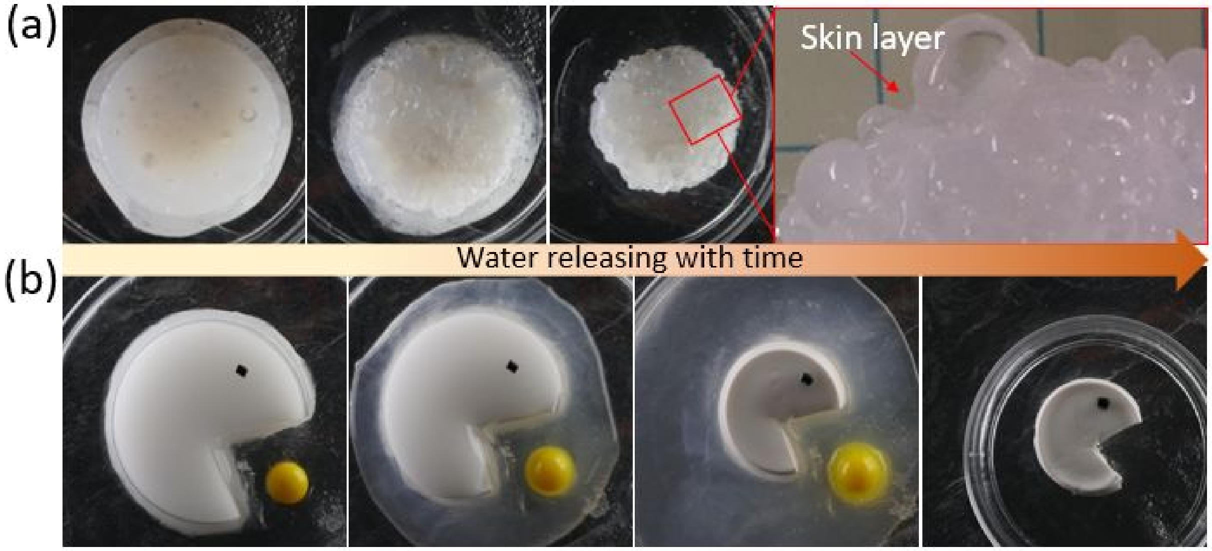

Fig. S9. Surface change of (a) C-PNIPAm and (b) L-PNIPAm upon heating above the LCST.

Fig. S9a shows that C-PNIPAm has a pronounced skin layer that inhibits water release *via* the formation of bubbles as the temperature is raised above the LCST. In contrast, the surface of L-PNIPAm does not exhibit a skin layer (**Fig. S9b**), as evident by the lack of surface bubbles.

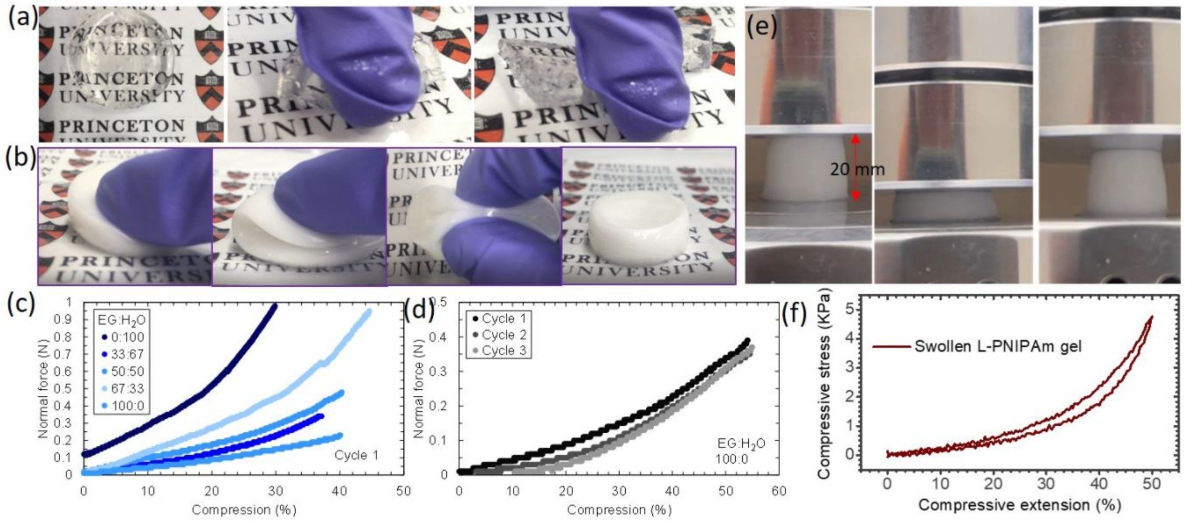

Fig. S10. (a) C-PNIPAm and (b) L-PNIPAm under compression. (c, d) Mechanical test of PNIPAm with different EG-water ratios before swelling in water. (e) Photographs of swollen L-PNIPAm with 20 wt.% NIPAm under a compression–decompression test cycle at a compressive strain of 50 %; and (f) the corresponding reversible compressive stress-strain curve.

C-PNIPAm is brittle and cannot sustain compression (**Fig. S10a**). In contrast, L-PNIPAm before and after swelling is soft and elastic (**Fig. S10b, e**). L-PNIPAm remained intact upon stress removal and recovered its original shape after 50 % compression (as seen in **Fig. S10 c, d, f**).

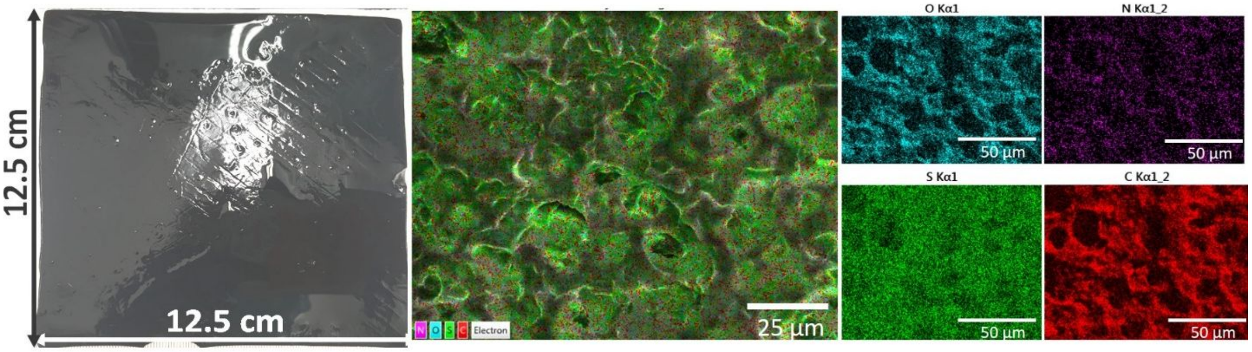

Fig. S11. Photographs and EDX mapping results of LSAG.

L-PNIPAm was modified with PDA and PSBMA to yield LSAG, a multifunctional gel with photoresponsive and antifouling properties. LSAG exhibited a dark color due to the presence of PDA atop the gel. Energy-dispersive X-ray (EDX) elemental mappings show the existence of S K-edge, C K-edge, N K-edge, and O K-edge elements, thus confirming the successful polymerization of PSBMA within LSAG.

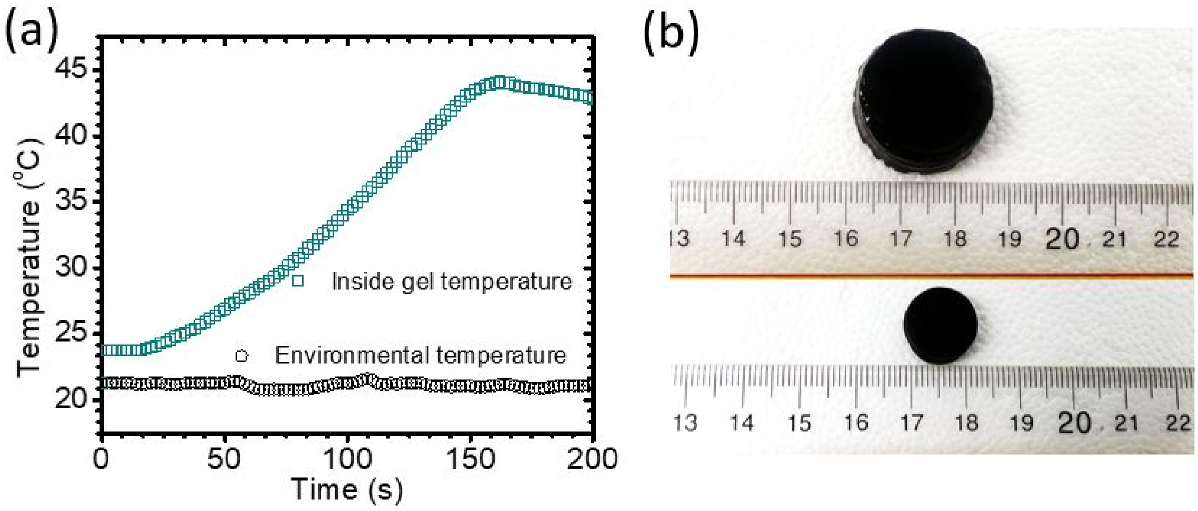

Fig. S12. Photothermal effect of LSAG under one sun irradiation. (a) The internal temperature change of a swollen LSAG. (b) Photographs of a swollen LSAG before and after one sun irradiation.

The photothermal conversion ability under sunlight irradiation was assessed by irradiating the LSAG with simulated sunlight of 1 kW m^{-2} (1 sun). Under this condition, the inner temperature of an LSAG increased with time and eventually reached ~ 43 °C (**Fig. S12a**). Benefiting from the solar thermal conversion property, the water-swollen gel can rapidly expel the stored liquid water when exposed to simulated sunlight with a noticeable volume change from the swollen state to a dried state (**Fig. S12b**).

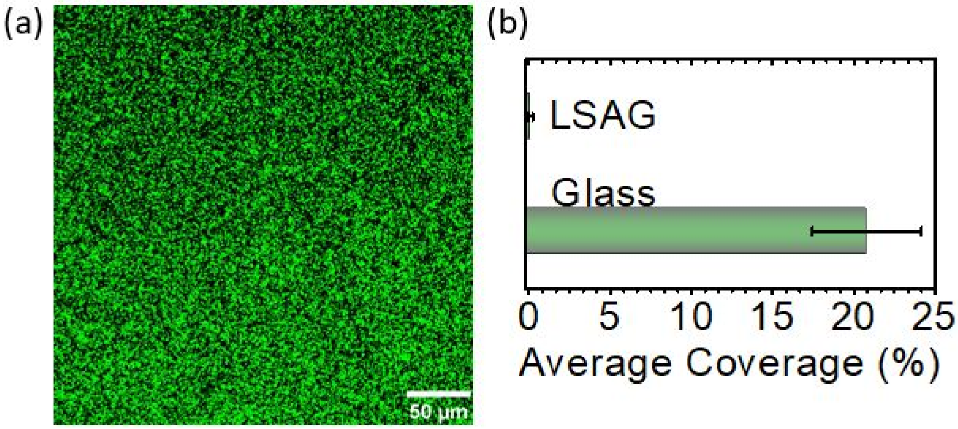

Fig. S13. (a) Fluorescence microscopy images of *E. coli* adsorption atop an L-PNIPAm/PDA gel surface with an average coverage of 20.8%. (b) Average coverage of *E. coli* atop a glass slide and LSAG after 24 hr of culture.

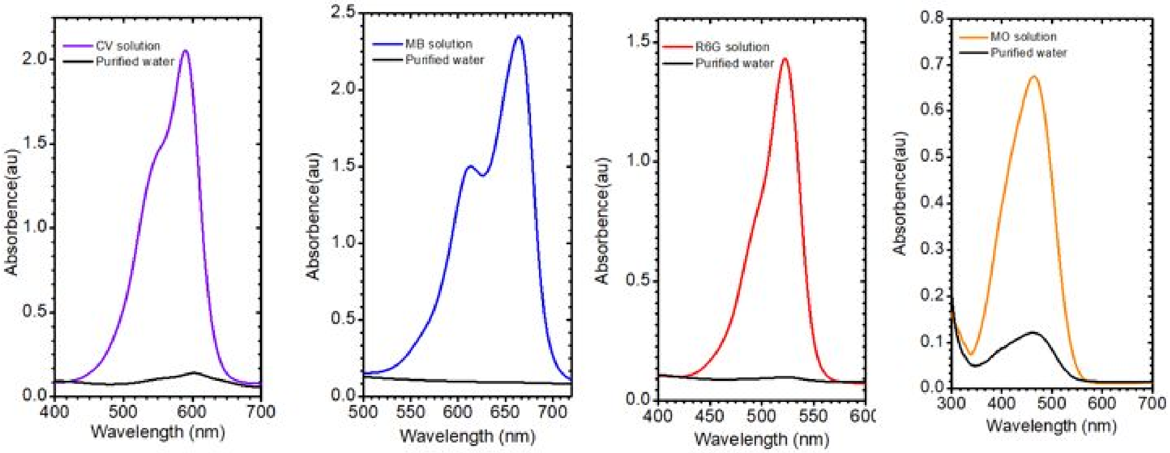

Fig. S14. UV-Vis measurement of the original dye solutions (CV, MB, R6G, MO) and purified water *via* LSAG.

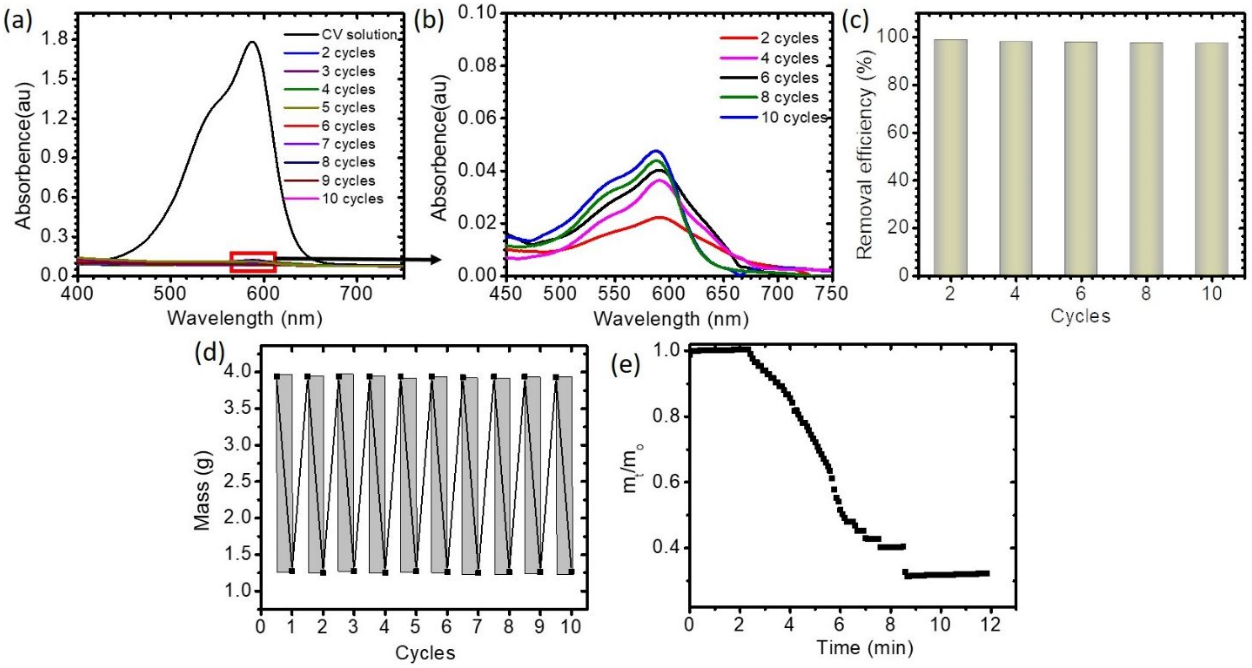

Fig. S15. Recycle stability of the LSAG for CV removal. (a, b) UV-Vis spectrum of the produced water from LSAG with different cycles. (c) Removal efficiency of LSAG. (d) The mass change during the cycling of LSAG; the shaded regions indicate that the LSAG was under 1 sun irradiation for 10 min. (e) The water production rate of LSAG during the decontamination process.

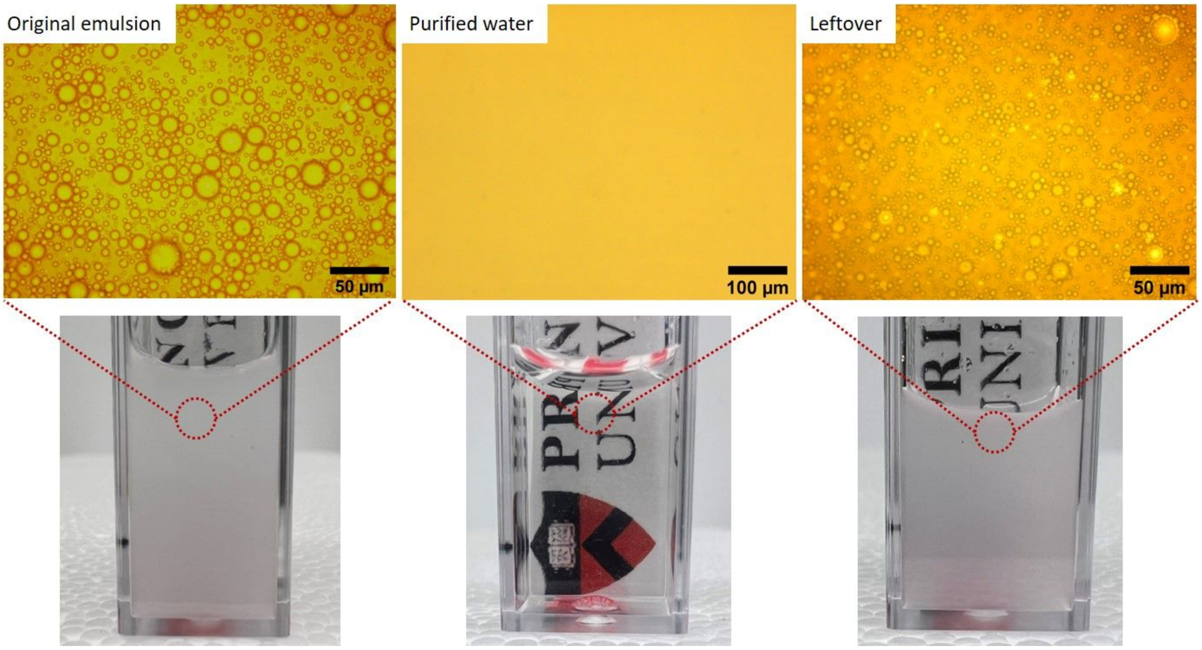

Fig. S16. Original SDS-stabilized olive oil-in-water emulsion and the purified water via LSAG.

The original olive oil-in-water emulsion is milky white and contains numerous oil droplets with diameters between ~ 5 - 20 μm. After purification *via* LSAG, the collected water is clear and free of visible oil droplets, whereas the residual emulsion remains turbid with many oil droplets.

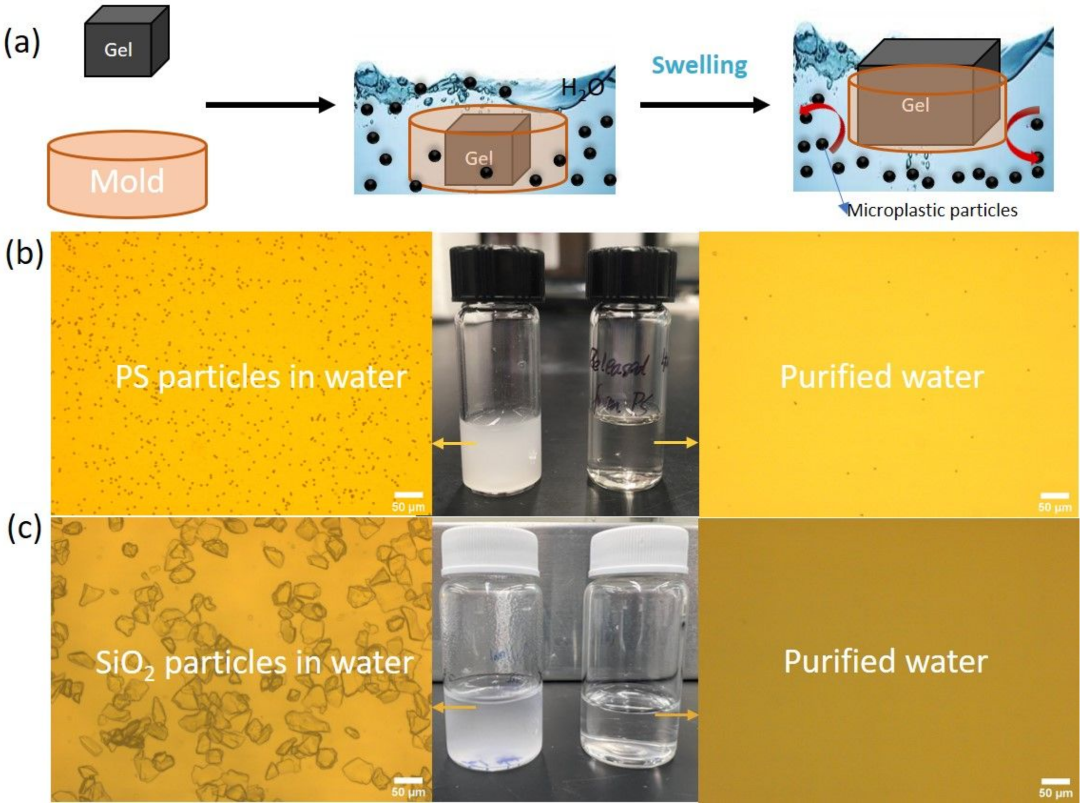

Fig. S17. (a) Purification process of water containing PS microplastic particles. (b) PS circular particles with a diameter of ~ 3.2 μm. (c) Irregular silica particles with a diameter of ~ 50 μm.

The purification process of water containing microplastic particles is shown in **Fig. S17a**. The microscopic images of the original microplastic particle suspensions and the LSAG-purified water are shown in **Fig. S17b, c.** The original microplastic suspensions are opaque, and the well-dispersed particles can be seen from the microscopic photographs. In the LSAG-produced water, no microplastic particles are observed.

Movie S1. The water diffusion test of conventional PNIPAm hydrogel and the loofah-like PNIPAm hydrogel.

Movie S2. The anti-oil fouling property of LSAG.

References

1 Sedlačík, T. S. *et al.* Preparation of tough double-and triple-network supermacroporous hydrogels through repeated cryogelation. *Chemistry of Materials* **32**, 8576-8586 (2020).
